# Supplementary material for: Flexible NAD+ Binding in Deoxyhypusine Synthase Reflects the Dynamic Hypusine Modification of Translation Factor IF5A
Source: Int J Mol Sci. 2020 Jul 31;21(15):5509. doi: 10.3390/ijms21155509 (PMC7432094; doi:10.3390/ijms21155509)
Supplement: Supplementary file 1 [file ijms-21-05509-s001.pdf]

International Journal of *Molecular Sciences*

Supporting Information for

Flexible NAD$^+$ binding in deoxyhypusine synthase reflects the dynamic hypusine modification of translation factor IF5A

Meirong Chen [1,2,†], Zuoqi Gai [1,†,‡], Chiaki Okada [1,§], Yuxin Ye[1,||], Jian Yu[1] and Min Yao[1,*]

1 Faculty of Advanced Life Science, Hokkaido University, Sapporo 060-0810, Japan；chenmr1987@163.com (M.C.); gaizuoqi@126.com (Z.G.); chiaki@hoku-iryo-u.ac.jp (C.O.); yeyx@pkusz.edu.cn (Y.Y.); yu@castor.sci.hokudai.ac.jp (J.Y.)
2 College of Food Science and Technology, Nanjing Agricultural University, Nanjing 210095, China
† These authors contribute equally to this work.
‡ The present address: School of Food Science and Engineering, Foshan University, Foshan 528225, China
§ The present address: School of Pharmaceutical Sciences, Health Sciences University of Hokkaido, Ishikari-Tobetsu, 061-0293, Japan
|| The present address: School of Chemical Biology and Biotechnology, State Key Laboratory of Chemical Oncogenomics, Peking University Shenzhen Graduate School, 518055 Shenzhen, China
* Correspondence:Correspondence: yao@castor.sci.hokudai.ac.jp; Tel./Fax: +81-11-706-4481.

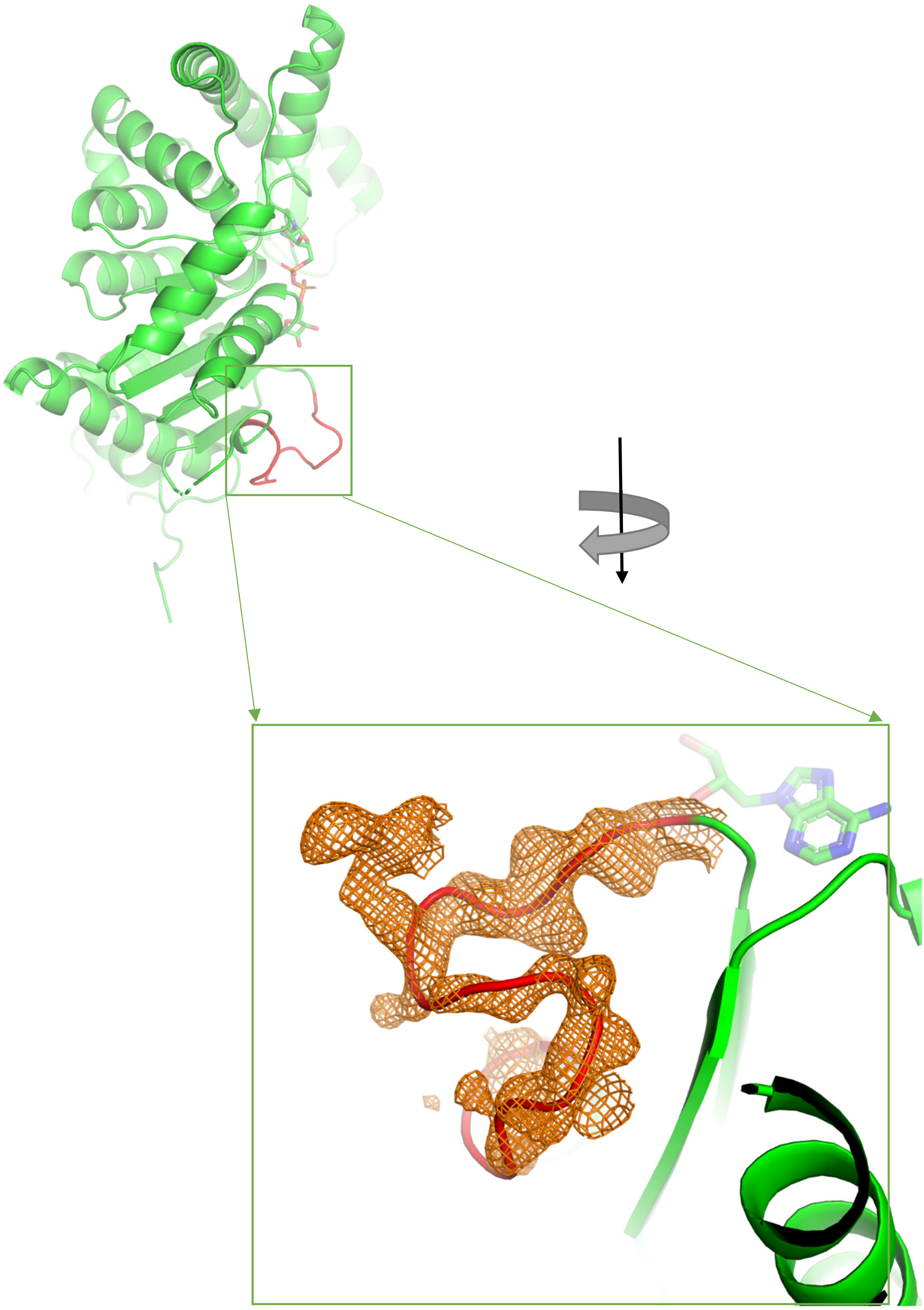

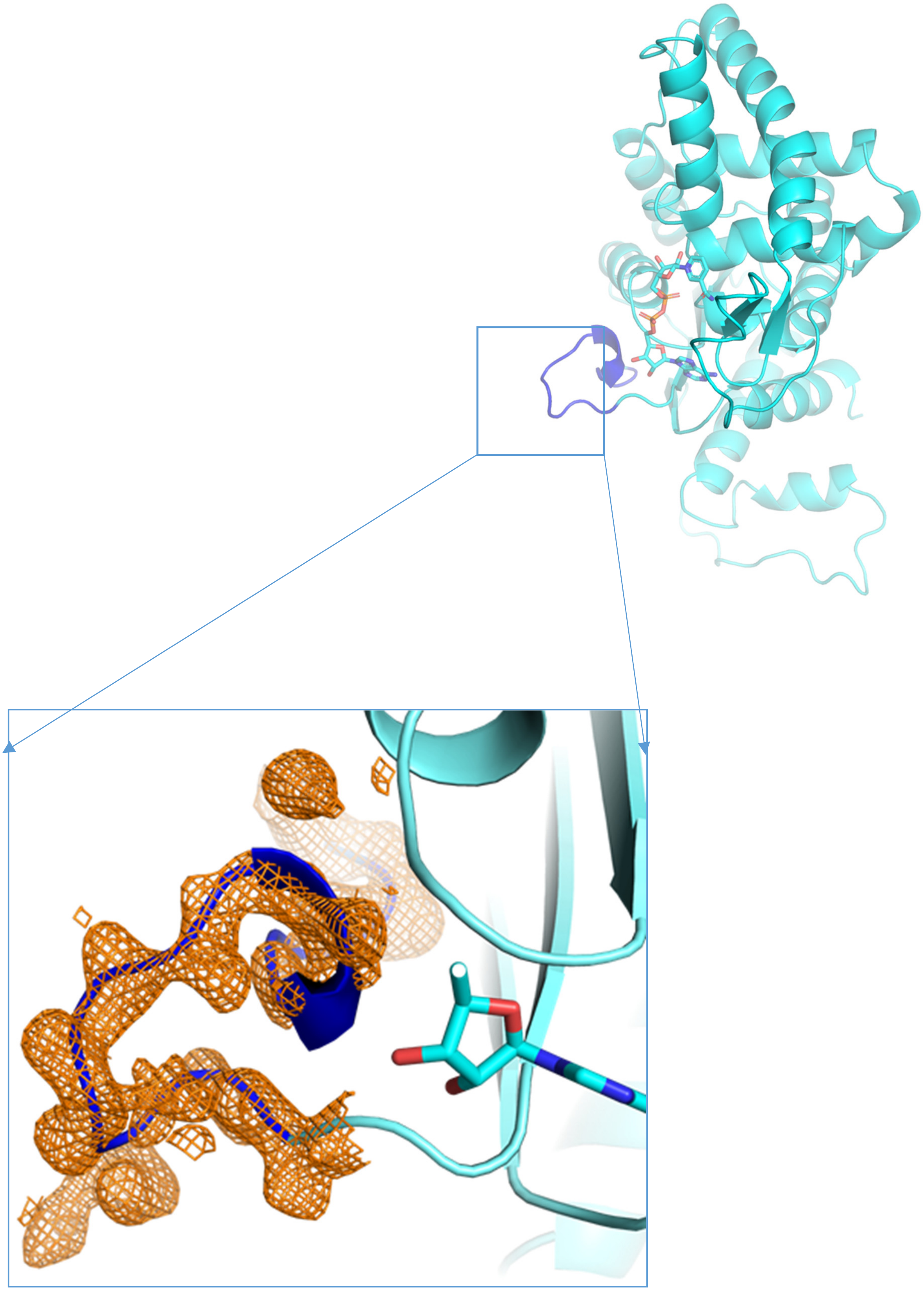

Figure S1. The loop region (V288-P299) in two different conformations (red and blue) in molA (green) and molD (cyan), respectively, with the 2*Fo-Fc* density map contoured at the 1σ level (0.2654e/Å^3) (orange).

(A)

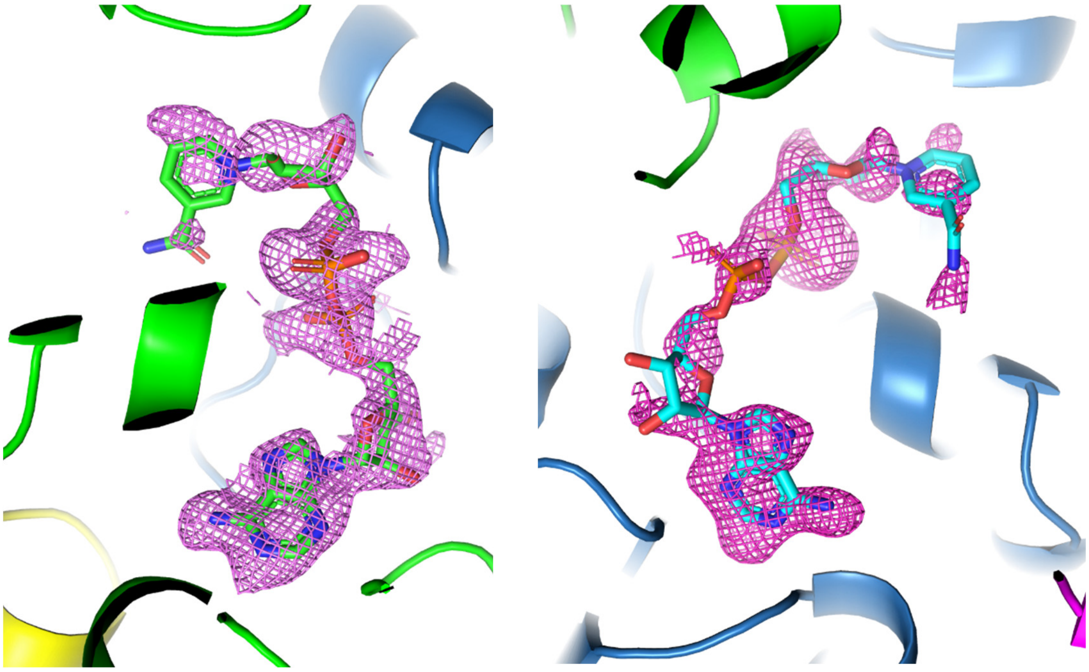

(A) **(B)**

Figure S2. The NAD⁺ molecules in molA (A) and molD (B) with the *Fo-Fc* omit map contoured at the 2σ level (purple).
